# A Novel Homozygous Mutation in the *COL4A4* Gene (Gly1436del) Causing Alport Syndrome Exposed by Pregnancy: A Case Report and Review of the Literature

**DOI:** 10.1155/2022/5243137

**Published:** 2022-01-04

**Authors:** Ulrich Jehn, Cornelie Müller-Hofstede, Barbara Heitplatz, Veerle Van Marck, Stefan Reuter, Hermann Pavenstädt, Britta George

**Affiliations:** ^1^Department of Medicine D, Division of General Internal Medicine, Nephrology and Rheumatology, University Hospital of Münster, Münster 48149, Germany; ^2^Institute of Human Genetics, University Hospital of Münster, Münster 48149, Germany; ^3^Gerhard Domagk Institute of Pathology, University Hospital Münster, Münster 48149, Germany

## Abstract

**Background:**

Alport syndrome results from a hereditary defect of collagen IV synthesis. This causes progressive glomerular disease, ocular abnormalities, and inner ear impairment. *Case Presentation*. Herein, we present a case of Alport syndrome in a 28-year-old woman caused by a novel mutation (Gly1436del) in the *COL4A4* gene that was not unveiled until her first pregnancy. Within the 29^th^ pregnancy week, our patient presented with massive proteinuria and nephrotic syndrome. Light microscopic examination of a kidney biopsy showed typical histological features of segmental sclerosis, and electron microscopy revealed extensive podocyte alterations as well as thickness of glomerular basement membranes with splitting of the lamina densa. One and a half years after childbirth, renal function deteriorated to a preterminal stage, whereas nephrotic syndrome subsided quickly after delivery.

**Conclusion:**

This case report highlights the awareness of atypical AS courses and emphasizes the importance of genetic testing in such cases.

## 1. Background

Alport syndrome (AS) is a hereditary disease that impairs the synthesis of type IV collagen. It clinically presents with progressive glomerular disease, ocular abnormalities, and sensorineural hearing loss [1]. Atypical manifestations include leiomyomatosis and vascular alterations [2].

AS is inherited either X-linked (XLAS, ∼ 80–85% of cases), autosomal dominant (ADAS, ∼ 5%), or autosomal recessive (ARAS, ∼ 10–15%). Mutations can affect the genes *COL4A3, COL4A4* (both located on chromosome 2, ADAS or ARAS), and *COL4A5* (located on the X-chromosome, XLAS), which encode *α*3, *α*4, and *α*5 chains of type IV collagen, respectively. To date, at least 1422 mutations causing AS have been reported [3].

The renal genotype-phenotype correlation of AS is variable, differs to some extent between males and females, and is among others dependent on the mode of inheritance (see below). The main clinical findings are microscopic hematuria, proteinuria, and progressive loss of renal function culminating in end-stage renal disease (ESRD) [4]. Heterozygous mutations in *COL4A3* or *COL4A4* usually present as thin basement membrane nephropathy without impairment of renal function [5].

In women, the first clinical manifestation of AS may appear as nephrotic syndrome during pregnancy, as pregnancy-related hyperfiltration may acutely worsen renal function [6, 7]. However, due to the unspecific and variably graded clinical manifestations and the possible progression towards a renal phenotype with histological patterns of more common glomerular entities associated with proteinuria, AS remains a highly under- or misdiagnosed entity [4].

Therapeutic options to slow down the progression of renal failure and proteinuria in AS are limited. They include the administration of angiotensin-converting enzyme-inhibitors (ACE-I), in second-line angiotensin-receptor blockers (ARBs) or aldosterone inhibitors [4], and strict treatment of arterial hypertension. Other therapeutical options such as antisense oligonucleotides or stem-cell administration are at an experimental stage [8]. An anti-inflammatory approach using bardoxolone methyl, which inhibits NF-*κβ*, is currently being investigated in a phase-3 clinical trial [9].

In this manuscript, we present a case of AS caused by a novel *COL4A4* mutation in a 28-year-old woman, which was fulminantly exposed by pregnancy.

## 2. Case Presentation

A 28-year-old woman from Syria presented with nephrotic syndrome starting during the 29^th^ week of her first pregnancy. Clinically, she showed anasarca with pleural effusions, ascites, and peripheral edema. At admission to our hospital, the patient showed normal blood pressure without antihypertensive medication. Initial laboratory findings included spot urine proteinuria of 17.2 g/g creatinine, hypoproteinemia of 3.8 g/dl [6.6–8.3 g/dl], serum albumin of 1.83 g/dl [3.9–5.0 g/dl], and serum creatinine of 1.4 mg/dl [<0.9 mg/dl] with an eGFR of 51 ml/min/1.73 m^2^ (CKD-EPI formula).

For an overview of the patient's baseline characteristics, see Table 1.

There were no preexisting diseases in her medical history. Unfortunately, data on the renal function prior to her pregnancy are not available. Medical history revealed that her only sibling, an older sister, who still lives in Syria, was also diagnosed with nephrotic syndrome, which progressed to ESRD and was treated with a kidney transplantation. Interestingly, the cause of ESRD in the patient's sister has not yet been identified, but the onset of the sister's kidney disease also manifested during pregnancy. Both parents, who also live in Syria, do not knowingly suffer from any renal disease to the knowledge of our patient. Nevertheless, they have not been tested for asymptomatic microhematuria and/or microalbuminuria.

Extensive diagnostic workup was performed. Among others, phospholipase A2- (PLA2-) receptor- and thrombospondin type 1 domain-containing 7A (THSD7A) antibodies were negative, as well as ANA, ANCA, and anti-GBM antibodies. Preeclampsia was excluded by negative soluble fms-like tyrosine kinase-1/placental growth factor (sFlt/PlGF) ratio.

Given the clinical presentation, steroid therapy with prednisolone 1 mg/kg body weight was commenced.

At 37 + 0 weeks of gestation, our patient was admitted to the obstetrics department for pharmacological induction of labor. Because cardiotocography revealed fetal distress and profuse vaginal bleeding caused by premature placental detachment during the opening period, an emergency C-section was performed. After transfusion of six red blood cell concentrates, the further postpartum course was unremarkable both for our patient and the newborn from a gynaecological and pediatric point of view, respectively.

**Figure 1:**
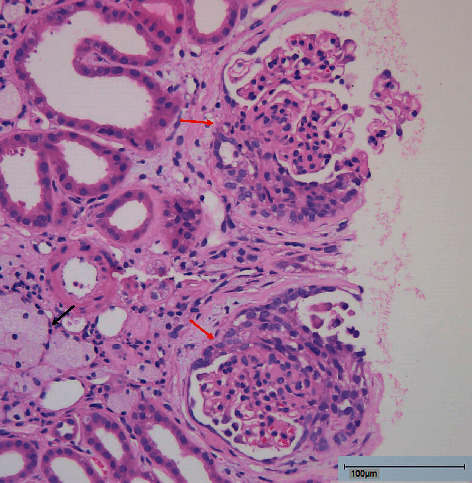
Photomicrography of the patient's kidney biopsy. It shows segmental sclerosis of 2 glomeruli (red arrows). The black arrow marks interstitial foamy macrophages, often seen in AS as well as in other types of chronic nephrosis. Bar: 100 *μ*m.

**Figure 2:**
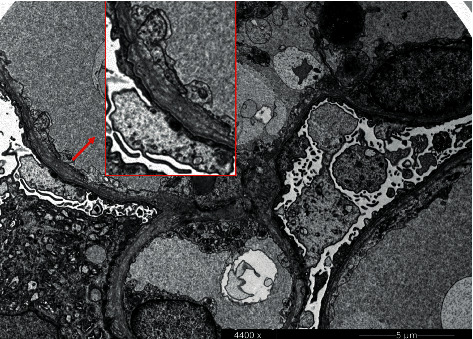
Electron micrography of the patient's kidney biopsy. It visualizes irregular thickness of glomerular basement membranes with splitting of the lamina densa (inset red box, 2-fold magnification) and severe podocyte foot process effacement. Bar: 5 *μ*m.

Two weeks after delivery, a kidney biopsy was performed. Light microscopic examination showed segmental sclerosis in 4 of 15 glomeruli (see Figure 1). Electron microscopy revealed extensive podocyte alterations with subtotal loss and effacement of the foot processes, as well as an irregular aspect of the glomerular basement membranes. Areas of remarkable thinning up to 130 nm alternated with areas with a multilamellar appearance with splitting of the lamina densa, the latter accounting for approximately 75% of the total basement membrane surface (see Figure 2).

Based on her medical history and actual findings suggestive of a hereditary AS, our patient underwent genetic evaluation.

Molecular genetic analysis proved an inframe deletion c.4307_4309del p.(Gly1436del) in the *Col4A4* gene causing loss of the amino-acid glycine. A wild-type allele was not detectable. Therefore, the mutation in our patient is probably present in a homozygous form. The patient believes that her parents are not consanguineous.

Treatment with ACE-I at the maximum dosage and good blood pressure control resulted in a significant decrease in proteinuria from 17.2 to 1 g/g creatinine three months postpartum, while eGFR further decreased to 37 ml/min/1.73 m^2^. During the following eighteen months, renal function deteriorated to a preterminal stage (eGFR 15 ml/min/1.73 m^2^), paralleled by a moderate increase in proteinuria to 2.4 g/g creatinine.

Of note, there is no clinical evidence of manifest ocular or inner ear involvement in our patient to date.

## 3. Discussion and Conclusions

Herein, we report the clinical manifestation of AS caused by the present inframe deletion c.4307_4309del p (Gly1436del) in the *Col4A4* gene, which is described here for the first time.

Based on the suggested homozygosity found in our patient and the fact that her parents were not noticeably affected, this constellation points towards an autosomal-recessive mode of inheritance.

At present, our patient's AS presents exclusively with significant renal involvement. Interestingly, in ARAS females, ocular and inner ear involvement is only present in a minority of patients [6, 10]. Basically, genotype-phenotype correlation in AS is variable. Most data addressing this question are available on the more common (85%) XLAS, whereas data on genotype-phenotype correlation in ARAS (15%) are sparse [11]. In XLAS, severe mutations as deletions, rearrangements, indels, and nonsense mutations are associated with earlier onset of ESRD compared to missense mutations (mean age 22.5 vs. 26.7 years) [11, 12]. Fitting to that, individuals with splice mutations or truncating mutations show a two-fold greater probability of developing ocular or hearing abnormalities [12].

In ARAS patients, mean age at ESRD onset was not found to be different for *COL4A3* or *COL4A4* mutations compared to those individuals with X-linked *COL4A5* mutations. Furthermore, Savige et al. did not detect differences for age at ESRD onset between homozygous and compound heterozygous mutations [11]. Congruent to XLAS, homozygous or compound heterozygous deletions, rearrangements, indels, and nonsense mutations enhance the onset of ESRD compared to those cases with missense mutations [11].

The patient's sister, who still lives in Syria, also suffered from nephrotic syndrome with initial presentation during pregnancy and has since received a kidney transplant. Although the underlying cause of the kidney failure has not been evaluated, the constellation strongly suggests that the sister may also have inherited AS.

Even if the patient denies a kinship relationship between her parents, this seems to be the most likely mode of inheritance [13]. In Middle Eastern countries, consanguinity is still a common phenomenon with 30.3% of marriages in urban and 39.8% in rural areas [14].

It is known that heterozygous mutations in the *Col4A4* gene are associated with benign forms of familiar hematuria, which are usually neither progressive nor lead to renal failure. This explains the bland parental medical history regarding renal disease [8]. However, neither our patient's parents nor her sister have been genetically evaluated in Syria.

In our patient, nephrotic syndrome occurred during the 29^th^ week of pregnancy with massive proteinuria and acute kidney failure. According to the literature, presentation of AS with these symptoms during the 29^th^–32^nd^ pregnancy week is typical for pregnant women with AS [7, 15].

The age at the time of clinical manifestation in the presented case of AS, which is highly suggestive for ARAS, was 28 years. Referring to the literature, this tends to be a relatively late onset of ARAS [10, 11].

Histologically, AS presented as segmental sclerosis in this case. In another case report of ARAS caused by a *Col4A4* mutation with manifestation during pregnancy, Drury et al. described the histological presentation of their patient as global segmental glomerulosclerosis with collapsing features, which might be caused by the additive effect of hyperfiltration during pregnancy [6]. Generally, the literature review shows an association of collagen IV mutations with segmental sclerosis [5, 16], which may lead to misdiagnosis of collagen IV nephropathies as FSGS [6].

The present case report is intended to raise awareness of atypical courses of AS and emphasizes the importance of medical family history and genetic testing in such cases.

## Figures and Tables

**Table 1:** Initial patient's characteristics and laboratory results.

Baseline characteristics	

Age	28 years
Pregnancy week	29^th^
Symptoms	Nephrotic syndrome anasarca
Medical history	Empty
Family history	Sister with unknown nephrotic ESRD also unveiled by pregnancy
Initial laboratory results	
Serum creatinine	1.4 mg/dl [<0.9 mg/dl]
eGFR	51 ml/min/1.73 m^2^
Protein/creatinine ratio	17.2 g/g creatinine
Total serum protein	3.8 g/dl [6.6–8.3 g/dl]
Serum albumin	1.8 g/dl [3.9–5.0 g/dl]
Initial blood pressure	130/70 mmHg

## Data Availability

The datasets analysed during this case report are not publicly available due to privacy policies. They are available from the corresponding author on reasonable request and with permission of the patient.

## Abbreviations

ACE-I: Angiotensin-converting enzyme-inhibitors
ADAS: Autosomal dominant Alport syndrome
ANA: Antinuclear antibodies
ANCA: Antineutrophil cytoplasmic antibodies
ARAS: Autosomal recessive Alport syndrome
ARB: Angiotensin-receptor blockers
AS: Alport syndrome
CKD-EPI: Chronic kidney disease epidemiology collaboration
ESRD: End-stage renal disease
FSGS: Focal segmental glomerulosclerosis
eGFR: Estimated glomerular filtration rate
GBM: Glomerular basement membrane
Indel: Insertion/deletion
NF-*κβ*: Nuclear factor “kappa-light-chain-enhancer” of activated B-cells
PlGF: Placental growth factor
PLA2: Phospholipase A2
sFlt: Soluble fms-like tyrosine kinase-1
THSD7A: Thrombospondin type 1 domain-containing 7A
XLAS: X-linked Alport syndrome.
